# Preliminary *Trichinella spiralis* Infection Ameliorates Subsequent RSV Infection-Induced Inflammatory Response

**DOI:** 10.3390/cells9051314

**Published:** 2020-05-25

**Authors:** Ki-Back Chu, Hae-Ahm Lee, Hae-Ji Kang, Eun-Kyung Moon, Fu-Shi Quan

**Affiliations:** 1 Department of Biomedical Science, Graduate School, Kyung Hee University, Seoul 02447, Korea; kbchu@khu.ac.kr (K.-B.C.); haedi1202@naver.com (H.-J.K.); 2 Medical Research Center for Bioreaction to Reactive Oxygen Species and Biomedical Science Institute, School of Medicine, Graduate School, Kyung Hee University, Seoul 02447, Korea; hae.ahm.lee@gmail.com; 3 Department of Medical Zoology, School of Medicine, Kyung Hee University, Seoul 02447, Korea; ekmoon@khu.ac.kr

**Keywords:** respiratory syncytial virus, *Trichinella spiralis*, antioxidant response, inflammation

## Abstract

Respiratory syncytial virus (RSV) infection affects the lives of neonates throughout the globe, causing a high rate of mortality upon hospital admission. Yet, therapeutic options to deal with this pulmonary pathogen are currently limited. Helminth therapy has been well received for its immunomodulatory role in hosts, which are crucial for mitigating a multitude of diseases. Therefore, in this study, we used the helminth *Trichinella spiralis* and assessed its capabilities for modulating RSV infection as well as the inflammatory response induced by it in mice. Our results revealed that RSV-specific antibody responses were enhanced by pre-existing *T. spiralis* infection, which also limited pulmonary viral replication. Diminished lung inflammation, indicated by reduced pro-inflammatory cytokines and inflammatory cell influx was confirmed, as well as through histopathological assessment. We observed that inflammation-associated nuclear factor kappa-light-chain enhancement of activated B cells (NF-κB) and its phosphorylated forms were down-regulated, whereas antioxidant-associated nuclear factor erythroid 2-related factor 2 (Nrf2) protein expression was upregulated in mice co-infected with *T. spiralis* and RSV. Upregulated Nrf2 expression contributed to increased antioxidant enzyme expression, particularly NQO1 which relieved the host of oxidative stress-induced pulmonary inflammation caused by RSV infection. These findings indicate that *T. spiralis* can mitigate RSV-induced inflammation by upregulating the expression of antioxidant enzymes.

## 1. Introduction

Respiratory syncytial virus (RSV) infection is a common cause of neonatal and infantile acute lower respiratory infection (ALRI) [1,2]. Currently, RSV-associated ALRI accounts for approximately 28% of all ALRI cases, and its mortality in children ranges from 13% to 22% [2]. Administering palivizumab, a monoclonal antibody targeting the RSV F protein can drastically reduce the rate of infantile hospitalization but it’s high cost and prescription limited to high-risk infants in most countries have necessitated the need for an effective licensed vaccine [3,4]. Some of the symptoms associated with severe RSV infection in infants involve profuse inflammation, airway edema, bronchitis, hypoxia, and tissue necrosis [3,5]. Oxidative stress is one of the underlying factors contributing to severe inflammatory response upon RSV infection. Specifically, RSV down-regulates nuclear factor erythroid 2-related factor 2 (Nrf2) through histone deacetylation and proteasomal degradation, which consequently inhibits the expression of antioxidant enzymes required to counteract the inflammatory response [6]. A causal relationship between ROS production and pulmonary inflammation has been established through previous studies, as pulmonary inflammation and the lung pathologies associated with RSV infection were attenuated through antioxidant treatment [7]. To this extent, a therapeutic option that regulates oxidative stress to maintain homeostasis would have a significant impact on RSV infection control.

Parasite-induced immunomodulatory responses have been reported to regulate several viral infections in mammalian hosts. Helminth-associated immunomodulation and its therapeutic potential in regulating inflammatory diseases have been garnering attention over the past few decades, with the United States Food and Drug Administration and the European Medicine Agency approving the development of drug products based on helminth or its eggs [8]. Even clinical trials involving helminth therapy for treating various diseases, such as multiple sclerosis, colitis, and Crohn’s disease, have been conducted [8,9].

In the case of the helminth *Trichinella spiralis*, the causative agent of trichinosis, only a handful of co-infection studies involving *T. spiralis* and viruses have been reported. Earliest *T. spiralis* co-infection studies documented to date demonstrated the possibility of viral transmission via *T. spiralis* larvae in animal models [10,11] and that its infection left the hosts more vulnerable to Japanese encephalitis virus infection, as a result of abrogated defense mechanisms [12,13]. However, recent *T. spiralis* co-infection studies have reported interesting findings. Multiple studies have confirmed the anti-inflammatory effect of *T. spiralis*, especially regarding airway inflammation [14,15,16,17]. *T. spiralis* infection has also attenuated influenza-associated pathologies in mice [18]. Its anti-inflammatory effects were further demonstrated in other organs involving various diseases. For example, *T. spiralis* infection attenuated collagen-induced arthritis through immunomodulation involving the programmed death 1 (PD-1) pathway [19] and alleviated 2,4,6-trinitrobenzene sulfonic acid (TNBS)-induced colitis in mice [20]. Moreover, the anti-arthritic effects of *T. spiralis*-derived antigens have also been documented [21]. Yet, not a single study has reported the effect of *T. spiralis* infection in regulating RSV-induced inflammation in the lungs. Identifying additional methods to limit RSV infection would make a significant contribution to public health and relieve some of the socioeconomic burden associated with it.

Here, we investigated the potential role of *T. spiralis* infection in modulating respiratory syncytial virus infection. Our results indicate that infection with *T. spiralis* ameliorates the inflammatory response in mice by upregulating the expression of antioxidant enzymes, which are down-regulated by RSV. The findings of the current study further contribute to the previous works and suggest that *T. spiralis* can regulate oxidative stress in hosts as a mechanism of immunomodulation.

## 2. Materials and Methods

### 2.1. Cell, Parasite, and Virus Preparation

HEp-2 cells were cultured in complete Dulbecco’s modified Eagle medium (DMEM; Welgene, Daegu, South Korea) supplemented with 10% fetal bovine serum, penicillin, and streptomycin for RSV A2 strain propagation and plaque assay. Briefly, a confluent monolayer of HEp-2 cells were infected with RSV A2 in serum-free DMEM at 0.1 MOI for 1 h, 37 °C, 5% CO_2_. After 1 h, media were aspirated and cells were incubated in fresh serum-free media at 37 °C, 5% CO_2_ for 2 days. Infected cells were harvested with a cell scraper and contents were centrifuged at 3000 rpm, 10 min, 4 °C to remove supernatants and other cellular debris. Infected cells were resuspended in serum-free media, sonicated, and centrifuged. The supernatant fraction containing the RSV was aliquoted and stored at −80 °C until use. Virus titer and protein concentrations were determined by plaque assay and Micro BCA protein assay (Thermo Fisher Scientific, Waltham, MA, USA). *T. spiralis* muscle larvae were maintained in Sprague-Dawley rats until use. Prior to the experiment, *T. spiralis*-infected rats were sacrificed and muscle tissues were digested in pepsin-HCl solution overnight at 37 °C. The next day, digested contents were filtered through a wire mesh to remove tissue debris, and sedimented larvae were repeatedly washed with deionized water. Muscle larvae were counted under the microscope before infection. Protein concentrations were determined using the Micro BCA protein assay kit (Thermo Fisher Scientific, Waltham, MA, USA) and antigens were stored at −20 °C until use.

### 2.2. In Vivo Experiment and Animal Ethics

Seven-week-old female Balb/c mice were purchased from NARA Biotech (Seoul, South Korea) and subdivided into four groups (*n* = 6 per group): uninfected (naïve), *T. spiralis* infection control (Ts), RSV infection control (RSV), *T. spiralis* and RSV co-infection (Ts-RSV). *T. spiralis* larvae were maintained in 6-week-old female Sprague-Dawley rats. Initially, mice in Ts and Ts-RSV groups were orally infected with 150 *T. spiralis* muscle larvae. On day 14 post-*T. spiralis* infection, mice in RSV and Ts-RSV groups were intranasally infected with 3 × 10^6^ plaque forming units (PFU) of RSV A2. On day 18, blood was collected and all of the mice were sacrificed. Mice from each group were divided into two groups (*n* = 3). The right lung lobes in the three mice were briefly washed in PBS to remove blood, snap-frozen in liquid nitrogen (LN_2_), and stored in −80 °C for protein and RNA acquisition. The left lobe, which was also serially washed in PBS, were homogenized and its supernatants were used for plaque assays and cytokine assays. From the remaining 3 mice in each group, the right lobes were used for bronchoalveolar lavage fluid (BALF) collection, while the left lobes were used for histopathological assessment. Animals were housed in an approved facility with a 12 h day and night cycle, as well as easy access to food and water ad libitum. All of the experimental procedures involving animals have been approved and conducted under the guidelines set out by Kyung Hee University IACUC.

### 2.3. Serum Collection and RSV-Specific Antibody Response Detection

Blood of mice was collected through the retro-orbital plexus puncture on day 18 immediately before sacrifice. Acquired blood samples were incubated briefly and centrifuged at 6000 RPM for 10 min. Sera were collected and stored at −20 °C until use. RSV antigen-specific antibody responses were detected using enzyme-linked immunosorbent assay (ELISA) as previously described [22]. Each well of the 96-well plates (SPL Life Sciences, Korea) was coated with 4 ug/mL of RSV A2 dissolved in 100 uL of carbonate buffer (pH 9.5) and incubated overnight at 4 °C. After blocking with 0.2% gelatin at 37 °C for 1 h, wells were incubated with sera (1:100 dilution in PBS) from each of the groups for 1 h at 37 °C. Horseradish peroxide (HRP)-conjugated IgG, IgM, and IgA (1:2000 dilution in PBS) purchased from Southern Biotech (Birmingham, AL, USA) were used as secondary antibodies. O-phenylenediamine substrate purchased from Sigma Aldrich (St. Louis, MO, USA) was dissolved in substrate buffer with H_2_O_2_ and 100 uL of these substrate solutions were added to each well. Reactions were stopped by adding dilute H_2_SO_4_ and colorimetric changes were assessed by measuring the OD_490_ values using EZ Read 400 microplate reader (Biochrom Ltd., Cambridge, UK).

### 2.4. Lung Viral Load Reduction

Lung viral load reductions were detected using plaque assay and quantitative reverse transcription-polymerase chain reaction (qRT-PCR). Plaque assays were performed by following the method previously described [23]. Briefly, 12 well plates were seeded with HEp-2 cells and serially diluted lung homogenate supernatants were inoculated into the confluent monolayer of cells for 1 h at 37 °C. Cells were overlaid with 1% noble agar and incubated at 37 °C, 5% CO_2_ for 3 days. Following the incubation period, agar overlays from each of the wells were gently removed for washing. Cells were incubated with the monoclonal anti-mouse RSV fusion protein (Merck Millipore, Burlington, MA, USA), then with the HRP-conjugated goat anti-mouse IgG (Southern Biotech, Birmingham, AL, USA). Plaques were visualized by staining with the stable diaminobenzidine (Invitrogen, Carlsbad, CA, USA) and quantified by determining the plaque-forming units (PFU) of viruses. For RT-qPCR, total RNA from infected murine lung tissues was extracted using the RNeasy Mini Kit (Qiagen, Venlo, Netherlands). Prime Script 1st strand cDNA synthesis kit (Takara, Otsu, Japan) was used for cDNA synthesis, which was then mixed with the SYBR master mix (New England Biolabs, MA, USA). Viral mRNA was quantified by measuring the level of RSV nucleocapsid (*N*) gene, which were normalized to the endogenous level of murine β-actin (*Actb*) gene as previously reported [24]. Reaction conditions were as follows: 95 °C for 2 min, then 40 cycles at 95 °C 15 s and 60 °C for 1 min. Primer sets for N gene and *Actb* are as follows: (forward: 5′-ctatggtgcagggcaagtga-3, reverse: 5-gaatcctgcttcaccaccca-3′; GenBank KT992094.1) and (forward: 5′-CCACCATGTACCCAGGCATT-3′, reverse: 5′-CGGACTCATCGTACTCCTGC-3′; GenBank NM_007393.5), respectively.

### 2.5. Inflammatory Cytokine and Bronchoalveolar Lavage Fluid Cell Influx Detection

Homogenized lung supernatants were collected and stored at −80 °C until use. IFN-γ and IL-6 pro-inflammatory cytokine levels from the homogenized lung supernatants were detected using the OptEIA IFN-γ and IL-6 kits following the manufacturer’s instructions (BD Biosciences, San Jose, CA, USA). Inflammatory cell influx in the bronchoalveolar lavage fluid (BALF) were quantified under the microscope using a hemocytometer. Collected BALF samples from the murine lungs were centrifuged at 1200 RPM, 4 °C, 3 min, followed by red blood cell lysis using RBC lysis buffer (Sigma Aldrich, St. Louis, MO, USA). After centrifugation, supernatants were aspirated and cells were resuspended in 100 uL PBS, then counted.

### 2.6. Histopathological Assessment of Murine Lung Tissues

On day 18, mice were sacrificed and the bronchus leading to the right lobes were clamped to prevent formalin entry. The entire left lung lobes were formalin-inflated for paraffin embedding. Embedded tissues were sectioned and stained with hematoxylin and eosin (H&E) and periodic acid-Schiff (PAS). Histopathological changes were blindly scored to determine the severity of pulmonary inflammation using the following criteria: (0: no inflammation; 1: thickened alveolar septae; 2: thickened lumen and peribronchioles; 3: cellular influx into vasculature and bronchioles; 4: severe inflammation).

### 2.7. Protein Expression Using Western Blot

Snap-frozen lung tissues were resuspended in protein lysis buffer and sonicated. Homogenates were vortexed briefly, incubated on ice for 10 min, for a total of 3 times. Samples were centrifuged at 13,500 RPM at 4 °C and supernatants were collected to determine protein concentration via Bradford assay. Proteins were separated on a 10% gel using sodium dodecyl sulfate-polyacrylamide gel electrophoresis (SDS-PAGE) and transferred on to a nitrocellulose membrane. After blocking with 5% skim milk, membranes were probed using catalase (CST 14097), p65 NF-κB (CST 6956), phospho-p65 NF-κB (CST 3033), NQO1 (CST 3187) monoclonal antibodies (Cell Signaling Technology, Danvers, MA, USA), Nrf2 polyclonal antibody (ab31163; Abcam, Cambridge, UK), superoxide dismutase 1 (SC-101523), and beta-actin (SC-47778) monoclonal antibodies (Santa Cruz Biotechnology, Dallas, TX, USA) and incubated overnight at 4 °C. HRP-conjugated anti-mouse IgG and anti-rabbit IgG were used as secondary antibodies, then expressions were detected on an x-ray film in the darkroom following enhanced chemiluminescence (ECL) exposure. Densitometry analyses of Western blot images were performed using the ImageJ software.

### 2.8. Statistical Analyses

All parameters were recorded for individual mice from each of the groups. Data sets were presented as mean ± SEM. Analyses were performed using GraphPad Prism 5 software (San Diego, CA, USA) and statistical significances were determined using one-way analysis of variance (ANOVA) with Tukey’s post-hoc test and two-tailed Student’s *t*-test.

## 3. Results

### 3.1. T. spiralis Infection Contributed to Mounting an Enhanced RSV-Specific Antibody Response

**Figure 1:**
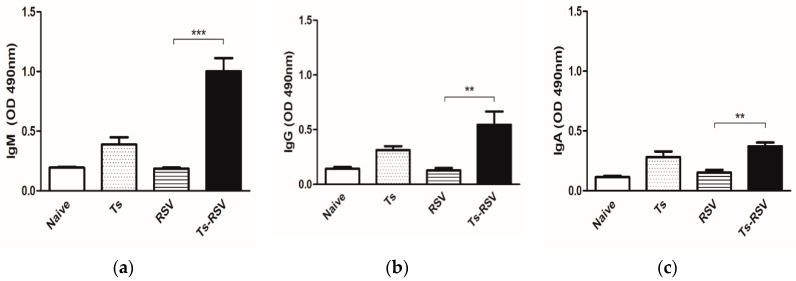
Enhancement of RSV-specific antibody responses can occur through pre-existing *T. spiralis* infection. Sera collected on day 18 prior to sacrifice were used to assess RSV-specific antibody responses. IgM (**a**), IgG (**b**), and IgA (**c**) levels in sera were determined by ELISA. Data are representative of three independent experiments presented as mean ± SEM, and statistical significance was determined using one-way ANOVA with Tukey’s *post hoc* analysis (** *p* < 0.01, *** *p* < 0.001).

RSV-specific antibody responses were strongly augmented by *T. spiralis* infection in mice. Notably, enhanced IgM (Figure 1a) and IgG (Figure 1b) antibody responses specific for the RSV antigen were detected from the sera of Ts-RSV and Ts mice. Similarly, enhanced IgA levels were also observed in the Ts-RSV and Ts mice (Figure 1c).

### 3.2. Preliminary T. spiralis Infection Impedes Viral Replication of RSV in the Lungs

**Figure 2:**
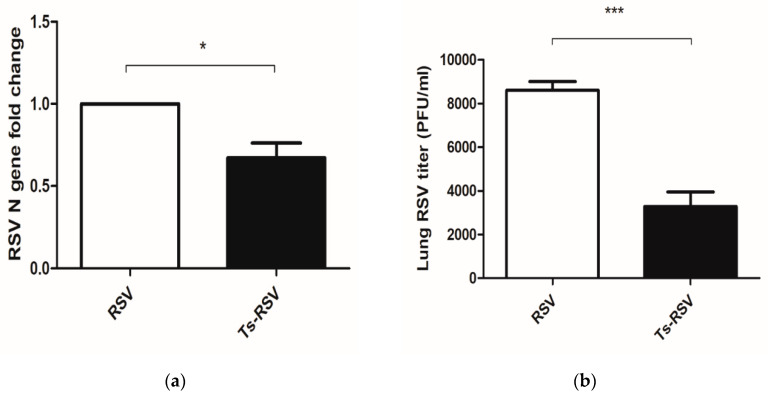
*T. spiralis* inhibited RSV replication in the lungs of mice. Of the 6 mice in each group, 3 mice were randomly sacrificed and their right lung lobes were snap-frozen in LN_2_ for protein and RNA extraction. The left lung lobe was homogenized and centrifuged to acquire supernatants, which were used for plaque assay and cytokine assays. RSV N genes in the acquired RNA samples of each group were quantified by RT-qPCR and results were normalized to endogenous *Actb* levels (**a**). Plaque assay was performed using the other lung fraction (**b**). Data are representative of three independent experiments presented as mean ± SEM, and statistical significance was determined using a two-tailed unpaired Student’s *t*-test (* *p* < 0.05, *** *p* < 0.001).

To assess whether enhanced antibody responses contributed to viral clearance in the lungs, lung virus titers were assessed by qPCR and plaque assay. Results of qPCR revealed an approximately 33% reduction in the lung viral load (Figure 2a), which were strikingly different from the 62% reduction observed in the plaque assay (Figure 2b). Nevertheless, findings are consistent as reductions in RSV viral loads in the lung tissues were observed.

### 3.3. T. spiralis Curtails Pulmonary Inflammation Triggered by RSV Infection

**Figure 3:**
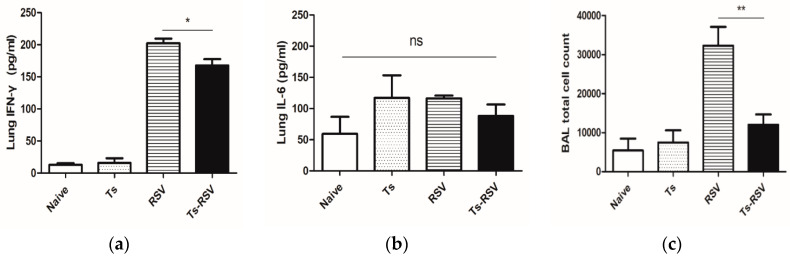
Pre-existing *T. spiralis* infection in mice attenuates RSV-induced pulmonary inflammation. Pro-inflammatory cytokine levels and inflammatory cell influx in the bronchoalveolar lavage fluid were quantified. Individual lung homogenates collected from the left lobe of 3 mice were used to assess IFN-γ (**a**) and IL-6 (**b**) levels. Bronchoalveolar lavage fluid was collected in 500 uL PBS from the right lobe of the remaining 3 mice by constricting the pulmonary blood vessels with a clamp (**c**). Cells were counted under the microscope after RBC lysis. Data are representative of three independent experiments presented as mean ± SEM, and statistical significance was determined using one-way ANOVA with Tukey’s *post hoc* analysis (* *p* < 0.05, ** *p* < 0.01, ns: *p* > 0.05).

To confirm if the reduced viral titers contributed to reduced inflammation in the lung tissues, pulmonary inflammatory cytokine levels and cellular influx in the BAL were assessed. Interferon-gamma (IFN-γ) expressions in lung tissues were slightly reduced in the Ts-RSV mice (Figure 3a) whereas interleukin-6 (IL-6) levels remained unaltered throughout all of the groups (Figure 3b). The inflammatory cellular influx in the bronchoalveolar lavage fluid was significantly less in the Ts-RSV group compared to the RSV control group (Figure 3c).

### 3.4. The Presence of T. spiralis Contributes to Alleviating Inflammatory Response Induced upon Subsequent RSV Infection

**Figure 4:**
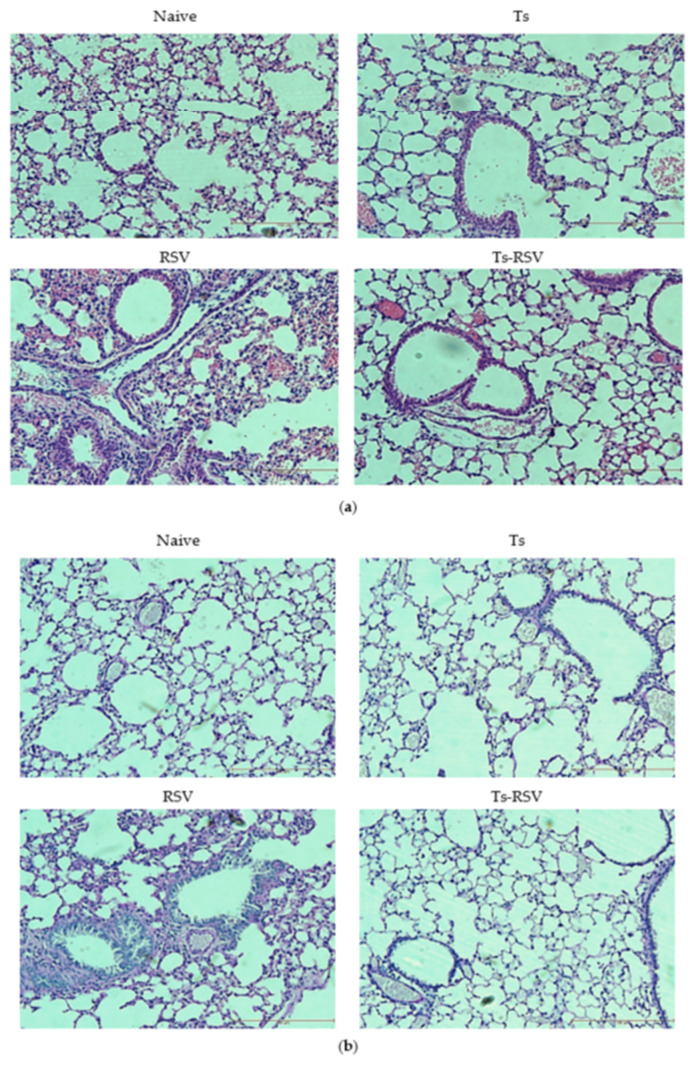

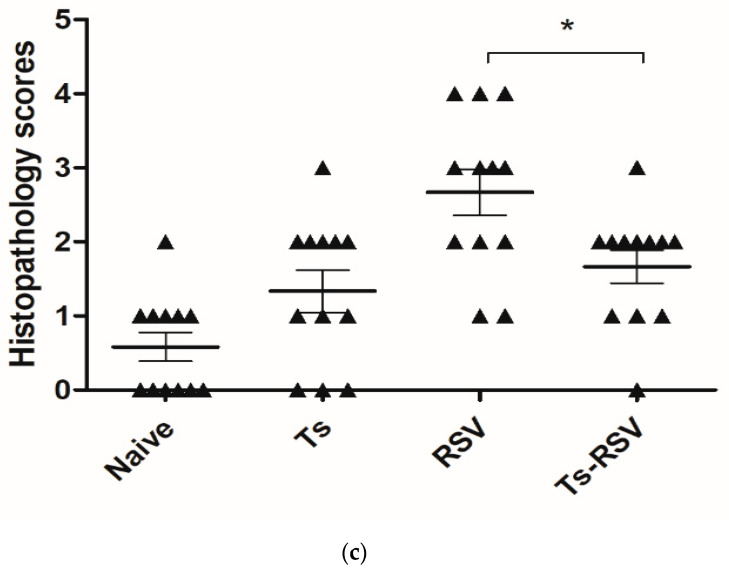
*T. spiralis* mitigates histopathological damage incurred by RSV infection. Left lobes of the 3 mice from each group were sent for histopathological assessment following formalin fixation. H&E and PAS stained lungs of mice from each group were visualized under the microscope at 200x magnification (**a**,**b**). Scale bar represents 100 μm. Histopathological changes were blindly scored based on the results of H&E and PAS staining (**c**). Statistical significance was determined using one-way ANOVA with Tukey’s *post hoc* analysis (* *p* < 0.05).

Lung histopathology examinations revealed that *T. spiralis* infection ameliorates inflammation induced by RSV infection. H&E staining results revealed that compared to naïve lung tissues, all of the infected groups possessed thickened septae lining the alveolar sacs, and multinucleated alveolar walls, which were most prevalent in the RSV infection group (Figure 4a). For PAS staining, lungs of *T. spiralis* control and Ts-RSV groups showed thickened alveolar walls with slight alveolar blebbing, whereas RSV infection incurred extensive blebbing, multinucleated alveolar walls, as well as mucin secretion (Figure 4b). Compared to the RSV control group, the inflammatory cellular influx into the lungs was significantly less prevalent in the Ts-RSV lungs (Figure 4c).

### 3.5. Antioxidant Enzyme Expressions were Enriched by T. spiralis Infection

**Figure 5:**
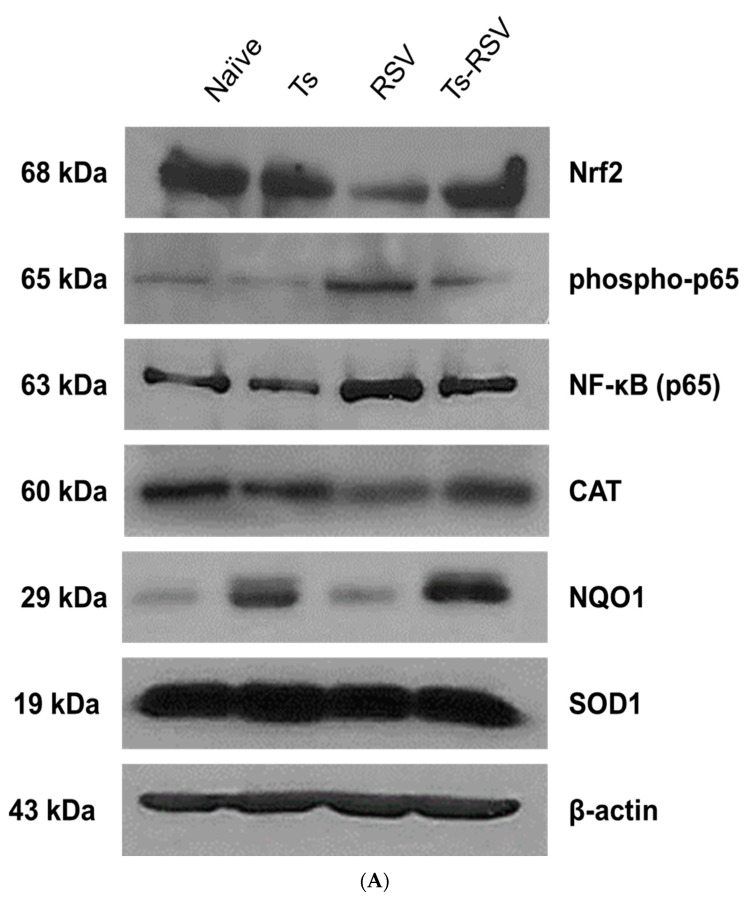

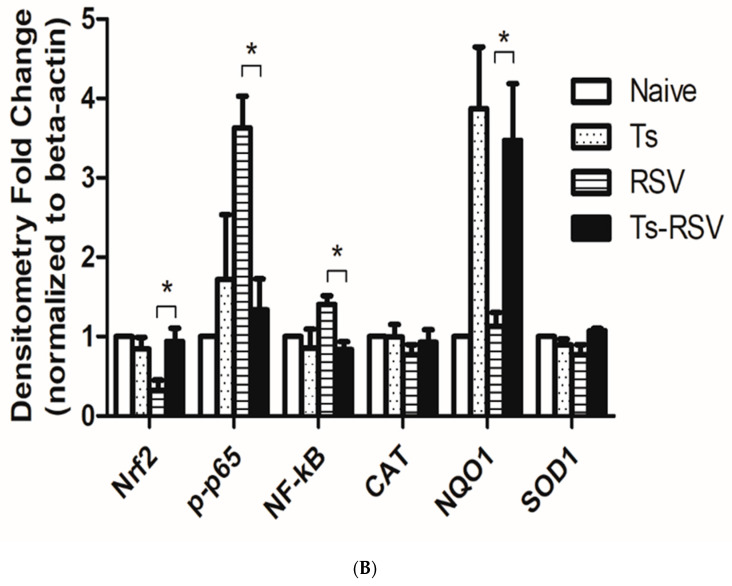
*T. spiralis* upregulates the expression of antioxidant enzymes in the lungs. Lung lysates were used to assess the level of Nrf2, phospho-p65, NF-κB, CAT, NQO1, and SOD1 expressions (**A**). RSV infection drastically reduced the expression of Nrf2 as expected, whereas *T. spiralis* infection augmented its expression. Compared to RSV control, a noticeable decrease in the inflammation associated NF-κB protein and phosphorylated form of its subunit phospho-p65 were observed. Densitometry analyses revealed significant differences in Nrf2, phospho-p65, and NF-κB expression between the RSV and Ts-RSV groups (**B**). Expression of Nrf2 gene downstream products NQO1, CAT, and SOD1 were enhanced in the Ts-RSV group. Of the three down-stream proteins, only NQO1 protein was enhanced to a significant extent whereas only partial increases were observed from the latter two. Data are representative of three independent experiments and statistical significance was determined using one-way ANOVA with Tukey’s *post hoc* analysis (* *p* < 0.05).

To assess the role of antioxidant expression in reducing the inflammatory response in the murine lungs, proteins involved in the inflammatory response have been investigated. Western blot results have revealed that single infection with *T. spiralis* alone had negligible effects on Nrf2, NF-κB, phospho-p65, although a noticeable increase in NQO1 was observed. On the contrary, a single infection with RSV resulted in diminished Nrf2 and partially diminished CAT and SOD1, while expressions of NF-κB and its phosphorylated form were greatly enhanced compared to control. In the Ts-RSV co-infected mice, the enhanced Nrf2 expression was paired with a partial increase in CAT and SOD1, with vastly upregulated NQO1 expression, while retaining low levels of NF-κB and phospho-p65 expressions (Figure 5A). Consistent with the representative images, densitometry analyses revealed no significant changes in antioxidant protein expressions except for NQO1. In the case of RSV, both NF-κB and phospho-p65 were drastically upregulated, while these two were down-regulated nearly two-fold in the Ts-RSV group. Although CAT and SOD1 were not strongly impacted in the Ts-RSV group, NQO1 expression was upregulated close to four-fold in comparison to RSV (Figure 5B).

## 4. Discussion

Immunomodulatory roles of parasites upon concomitant infection with another pathogen have been widely reported and this led us to inquire whether regulating pulmonary inflammation induced by RSV using the parasite *T. spiralis* was feasible. We addressed this question by co-infecting mice with *T. spiralis* and RSV, a notorious pulmonary virus inducing potent inflammatory responses in hosts. Our study demonstrated that *T. spiralis* infection can protect the mice from subsequent RSV infection by enhancing the antioxidant enzyme expression, which in turn limits the inflammatory response incurred by RSV.

As expected, sufficient levels of RSV-specific antibody responses were not observed in RSV control mice since viral infections only lasted four days. Contrary to the previous findings which reported extremely low levels of RSV-specific IgM response appearing around four days post-infection (dpi) [25,26], the increase in IgM response observed from the RSV control was negligible in comparison to the naïve group. However, consistent with the previous reports, IgA was partially increased in the RSV control mice compared to naïve while IgG levels remained unaltered [25,26]. Interestingly, our findings revealed that *T. spiralis*-specific antibodies are capable of cross-reacting with RSV, thereby conferring an enhanced antibody response against subsequent RSV infection. Nonetheless, it is noteworthy to mention that antibody cross-reactivity does not imply guaranteed virus neutralization, a key factor contributing to antiviral response. A study reported that serum samples from patients with prior exposure to the Dengue virus failed to effectively neutralize the Zika virus despite demonstrating potent cross-reactivity [27].

Although rare, several studies have documented the cross-reactivity between parasite and viral antigens. For instance, administering *Onchocerca volvulus* secretory protein as an adjuvant generated cross-reactive antibodies and conferred better protection against influenza virus infection than an inactivated influenza vaccine alone [28]. Several investigations outlining the presence of cross-reactivity between human T cell lymphotropic virus type I and *Plasmodium falciparum* have been reported [29,30]. In our previous investigation, we observed that an antibody generated against RSV can cross-react with the excretory-secretory proteins of *T. spiralis* [31]. Based on these findings, it is likely that antigenic cross-reactivity between *T. spiralis* and RSV exists as strongly enhanced RSV-specific IgM, IgG, and IgA antibody responses were observed in Ts and the Ts-RSV mice. These antibody responses may have contributed to the lessened lung viral loads or low RSV N gene copy numbers as observed in Ts-RSV mice.

Earlier studies have delineated the persistence of NF-κB following RSV infection, and that phosphorylation of its p65 subunit is an essential process contributing to NF-κB activity [32,33]. RSV infection was reported to trigger phosphorylation of the p65 subunit at S536, a crucial process involved in increasing NF-κB transactivation [34]. Consistent with the literature findings, RSV infection enhanced the protein expressions of NF-κB and phosphorylated p65 which were strongly down-regulated by *T. spiralis* in Ts-RSV mice. Our results revealed that *T. spiralis* infection can modulate the host immune response to cope with inflammatory virus infection in the lungs by regulating the reactive oxygen species levels. The p65 subunit involved in the NF-κB signaling has been documented to antagonize the Nrf2 expression by limiting the available CREB-binding protein (CBP), thereby suppressing the downstream expression of antioxidant enzymes [35,36]. This inhibitory mechanism contributed to the suppression of NF-κB in the Ts-RSV mice, whereas the reverse was the case for the highly inflamed lung tissues of RSV control mice, as demonstrated by inhibited Nrf2 expression. Helminthic parasites have evolved a mechanism to cope with reactive oxygen species generated through cellular metabolism, especially during their growth phase, as well as from the host immune effector cells [37,38]. Recent epigenetic investigations involving *T. spiralis* has revealed that antioxidant enzyme expressions were highly enriched throughout multiple stages of its life cycles [39]. Based on this finding, we speculate that these enriched antioxidant enzymatic expressions, which were present before the RSV infection, negated the free radical damage induced by RSV infection and thereby limited the severity of the disease onset. Several investigations have revealed the importance of antioxidant response and viral inhibition in various viruses. The protective role of Nrf2 can also be applied to RSV infection, as genes dependent on its expression are required for antiviral response and further limiting viral replication [40,41]. Evidently, increased expression of catalase and its expression have also been documented to confer protection against experimental RSV infection, which further validates the findings of our works [42]. In the current study, changes in CAT and SOD1 expressions in the Ts-RSV mice were negligible in comparison to RSV control, which indicates that these proteins only play a minor role, if not none, for ameliorating RSV-induced inflammation. Yet, NQO1 expressions in Ts and Ts-RSV lungs were increased nearly three-fold in comparison to the naïve and RSV control. Multitudes of studies have reported the importance of NQO1 for maintaining mitochondrial membrane potential, which consequently suppresses excessive oxidant generation [43]. The enhanced NQO1 expression has also been associated with increased superoxide scavenging, which alleviates the oxidative stress generated in cells [44]. From this, *T. spiralis*-induced NQO1 overexpression may have been the primary factor contributing to negating RSV-associated pulmonary damage.

Interesting results have been reported from multiple co-infection studies involving RSV. Although extremely limited in number, similar findings to the present study have been reported using different helminths. For example, RSV-induced pathologies were ameliorated by the enteric parasite *Heligmosomoides polygyrus* [45]. The filarial nematode *Acanthocheilonema viteae*-derived serine protease inhibitor successfully inhibited RSV-associated lung pathologies and inflammation in mice [46]. Histopathological assessment of lung inflammation was consistent with the cytokine expression and cellular influx in the BALF. As expected, severe pulmonary inflammation in the RSV-infected mice was observed, which were also present to a lesser degree in the *T. spiralis* infection group and the Ts-RSV group. In the case of Ts-RSV group, we speculate that the enrichment of antioxidant expression, paired with inhibition of NF-κB, contributed to limiting the severity of inflammation. In contrast to the works of McFarlane et al. [45], no significant differences in IL-6 were found whereas IFN-γ levels in Ts-RSV mice were significantly diminished, which may imply potentially different mechanisms involved in regulating RSV infection between *H. polygyrus* and *T. spiralis*. Although the role of antioxidant response has been demonstrated here, the exact mechanism of action underlying RSV inhibition by *T. spiralis* remains elusive and unraveling this phenomenon is of strong interest for future works. The occurrence of pulmonary inflammation in the Ts group was as expected, which can be explained using its life cycle. When the newborn larvae migrate to the highly oxygenated skeletal muscle via the circulatory and lymphatic system, it also bypasses the lungs which cause inflammation during this process [47,48,49]. Larvae exit the circulatory system and begin encysting in the muscles two weeks post-infection, as supported by drastically dwindling larvae counts observed from various organs and the blood around 13 dpi [50]. Since RSV infection occurred 14 days after *T. spiralis* infection, most of the larvae have encysted in the skeletal muscle while extremely few larvae may remain in the peripheral blood. Although investigating the anatomical distribution of the parasite and its implications for inducing immunomodulatory effect is beyond the scope of this study, it is highly plausible that *T. spiralis*-induced immunomodulation can occur regardless of the parasite’s location. Accordingly, the anti-inflammatory effects of *T. spiralis* have been observed from both intestinal and muscle phase larvae as well as their extracts [17,18,51].

Several investigations using different helminths have presented interesting results, which signify the importance of excretory-secretory products from helminths for suppressing various diseases [52,53,54,55,56]. In line with this notion, studies involving the *T. spiralis* ES antigen have revealed a similar phenomenon where its usage evoked anti-inflammatory responses [57,58,59,60]. Based on these previous investigations, one potential strategy to apply our findings would be to design a small prototype molecule analogous to that of the *T. spiralis* antigen that holds anti-inflammatory function. Applying this concept enabled the synthesis of a prototype drug analogous to ES-62 of *A. viteae*, which effectively suppressed the development of collagen-induced arthritis in a murine model [61]. To this extent, the application of this concept may yield promising results for limiting the severity of RSV infection.

In conclusion, the present study demonstrated that pre-existing infection with *T. spiralis* can inhibit subsequent RSV infection by upregulating antioxidant enzyme activity. Our findings demonstrated that *T. spiralis* infection enhanced RSV-specific antibody responses and suppressing viral replication. By down-regulating NF-κB and upregulating Nrf2 expression, mice with pre-existing *T. spiralis* infection experienced lesser inflammatory cytokines and inflammatory cellular influx in the lungs. Findings herein provide additional scientific evidence of the helminthic regulation of viral pathogens and outline its potential for use in inflammatory diseases.
